# Mesenchymal stem cells and their secreted molecules predominantly ameliorate fulminant hepatic failure and chronic liver fibrosis in mice respectively

**DOI:** 10.1186/s12967-016-0792-1

**Published:** 2016-02-09

**Authors:** Biao Huang, Xixi Cheng, Huafeng Wang, Wenjing Huang, Zha la Ga hu, Dan Wang, Kai Zhang, Huan Zhang, Zhenyi Xue, Yurong Da, Ning Zhang, Yongcheng Hu, Zhi Yao, Liang Qiao, Fei Gao, Rongxin Zhang

**Affiliations:** Department of Immunology and Research Center of Basic Medical Science; Tianjin Key Laboratory of Cellular and Molecular Immunology; Key Laboratory of Immune Microenvironment and Diseases, Ministry of Education of China, Key Laboratory of Hormones and Development (Ministry of Health), Metabolic Diseases Hospital and Tianjin Institute of Endocrinology, Tianjin Medical University, Qi Xiang Tai Road No.22, Tianjin, 300070 China; School of Life Science, Shanxi Normal University, Linfen, Shanxi Province China; Department of Cell Biology, Logistic College of CAPF, Tianjin, China; Department of Orthopaedic Oncology, Tianjin Hospital, Tianjin, China; Storr Liver Unit, Westmead Millennium Institute, The Western Clinical School of the University of Sydney, Westmead, NSW Australia; State Key Laboratory of Reproductive Biology, Institute of Zoology, Chinese Academy of Sciences, Beijing, 100101 China

**Keywords:** Liver failure, MSC-CM, Hepatic stellate cells, Macrophages, CD4^+^ T cells

## Abstract

**Background:**

Orthotopic liver transplantation is the only effective treatment for liver failure but limited with shortage of available donor organs. Recent studies show promising results of mesenchymal stem cells (MSCs)-based therapies.

**Methods:**

We systematically investigate the therapeutic effects of MSCs or MSC-conditioned medium (MSC-CM) in ameliorating fulminant hepatic failure (FHF) and chronic liver fibrosis in mice. In addition, extensive flow cytometry analysis of spleens from vehicle and MSC- and MSC-CM-treated mice was applied to reveal the alteration of inflammatory state.

**Results:**

In FHF model, MSCs treatment reduced remarkably the death incidents; the analysis of gross histopathology showed that control livers were soft and shrunken with extensive extravasated blood, which was gradually reduced at later time points, while MSC–treated livers showed gross pathological changes, even 24 h after MSC infusion, and hematoxylin and eosin staining revealed dramatical hepatocellular death with cytoplasmic vacuolization suppressed by MSCs treatment; flow cytometry analysis of total lymphocytes showed that macrophages (F4/80) infiltrated into control livers more than MSC-treated livers; by contrast, MSC-CM partially ameliorates FHF. In chronic liver injury model, MSC and MSC-CM both suppressed fibrogenesis and necroinflammatory, and the later was better; activation of hepatic stellate cells (α-SMA) was inhibited; glycogen synthesis and storage (indicated by periodic acid-Schiff -staining) was improved; liver regeneration (Ki67) was promoted while liver apoptosis (TUNEL) was reduced. In the in vitro, MSCs promote macrophage line RAW264.7 apoptosis and MSC-CM promotes apoptosis and inhibits proliferation of HSC line LX-2. We also found that MSCs and MSC-CM could improve spleen; MSC-CM increased levels of Th2 and Treg cells, and reduced levels of Th17 cells, whereas levels of Th1 cells were unchanged; comparatively, MSC treatment did not affect Th17 and Treg cells and only slightly alters inflammatory state; MSC and MSC-CM treatment both substantially down-regulated macrophages in the spleens.

**Conclusion:**

Both MSCs and MSC-CM exert therapeutic effects by acting on various key cells during the pathogenesis of FHF and chronic fibrosis, stimulating hepatocyte proliferation and suppressing apoptosis, down-regulating infiltrating macrophages, converting CD4^+^ T lymphocyte system into an anti-inflammatory state, and facilitating hepatic stellate cell death.

**Electronic supplementary material:**

The online version of this article (doi:10.1186/s12967-016-0792-1) contains supplementary material, which is available to authorized users.

## Background

For most animals, the liver is the most important metabolic organ, and end-stage liver failure is a potentially life-threatening state that is frequently accompanied by severe complications. Liver failure includes FHF and chronic liver fibrosis, which can further deteriorate into cirrhosis and hepatoma. Orthotopic liver transplantation is currently the most effective treatment, but its use is limited due to a shortage of available donor organs, high costs, and requirements for life-long immunosuppression [1, 2]. Therefore, recent studies have focused on the therapeutic effects of different types of stem cells, which can improve liver function via distinct mechanisms [3, 4].

MSCs, a specific type of adult stem cell isolated from compact bone, can differentiate into multi-lineage cells [5]. Moreover, MSCs naturally support homeostasis by secreting soluble factors, including trophic molecules and cytokines, which indirectly regulate the immune system [6]. Recent studies show that MSCs down-regulate pro-inflammatory cells via different mechanisms in autoimmune diseases [7, 8]. That is, different MSC therapies can alleviate the negative outcomes of injury and diseases via either interactions between MSC and various target cells [9–11] or MSC-secreted soluble factors [12]. However, differences between MSC therapies for liver failure have not been fully explored.

In the present study, we systematically studied the effects of MSCs and MSC-CM containing various MSC-secreted soluble factors on FHF and chronic liver fibrosis in mice, focusing on CD4^+^ T lymphocytes, macrophages, and hepatic stellate cells (HSCs), which play important roles in the pathogenesis of liver failure. We showed that both MSC and MSC-CM treatment improved recovery from liver failure, although there were differences in their modes of action. That is, MSCs ameliorated FHF by interacting with various inflammation-relevant cells, thereby achieving immunosuppression and promoting survival, whereas MSC-CM ameliorated chronic liver fibrosis by down-regulating inflammatory responses and promoting HSC apoptosis.

## Methods

### Mice

Two-to-three-week-old female ICR mice and six-to-eight-week-old female ICR or C57BL/6 mice weighing 20-25 g were purchased from the Laboratory Animal Center, Academy of Military Medical Sciences (Beijing, China) and were housed in conventional cages. All experiments in this study were performed in accordance with the Academy of Military Medical Sciences Guide for Laboratory Animals.

### Isolation and culture of bone-derived MSCs

MSCs from murine compact bone were isolated and culture-expanded as described in previous report [13]. Phenotype and multipotent stem cell characteristics of infused MSCs were analyzed (Additional file 1: Figure S1). The details are placed in Additional file 1.

### Preparation of MSC-CM

Conditioned growth medium was concentrated 25-fold through ultrafiltration units (Millipore, Bedford, MA) with a 3-kDa cutoff [14]. The details are placed in Additional file 1.

### Animal model and treatment protocols

The details are placed in Additional file 1.

### Induction of FHF

FHF was induced by a single dose of thioacetamide (TAA; dissolved into 40 μg/μl with sterile PBS) injected intraperitoneally. After 5 h of TAA injection, 200 μl MSC-CM or 1 × 10^6^ MSCs and 200 μl PBS solution (vehicle control) was infused into the tail vein.

### Induction of chronic liver fibrosis

To induce liver fibrosis, mice received 12 consecutive intraperitoneal injections (1 μl/g body weight) of CCl_4_: olive oil (1:1) twice per week for 6 weeks. 1 × 10^6^ MSCs were infused into the tail vein, or 200 μl MSC-CM were infused into tail vein twice per week for three consecutive weeks at the sixth week of CCl_4_ injections.

### Immunohistochemistry

Various immunohistochemistry staining are performed as described in previous report [14]. The details are placed in Additional file 1.

### Statistical analysis

Results are expressed as the mean ± standard deviation. Statistical significance was determined by a two-tailed Student t test. Survival was analyzed using the Kaplan–Meyer product limit estimate and compared with the log-rank test.

## Results

### MSCs improve gross and microscopic liver histopathology and prolong survival of mice with FHF

**Fig. 1:**
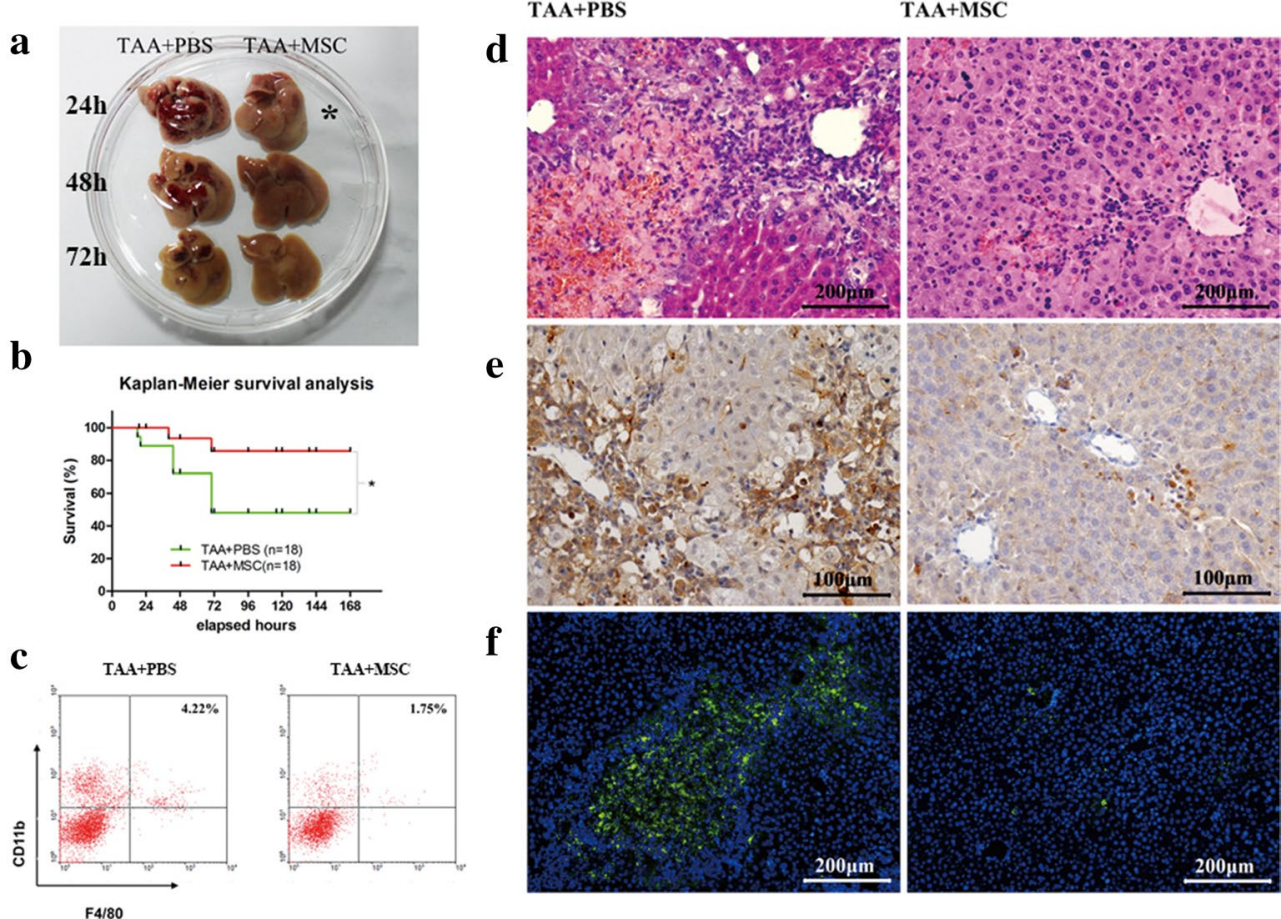
MSC infusion promotes survival, improves gross and microscopic histopathology in TAA-induced FHF. **a** MSC treatment substantially improved the gross histopathological appearance of TAA-stimulated livers with reduction of extravasated blood. *Asterisk* marks the changes occurred to liver 24 h after MSC infusion. **b** Kaplan–Meier survival analysis of mice with TAA-induced FHF. **c** Flow cytometry analysis of macrophages. **d** HE staining **e** F4/80 immunohistochemistry **f** CD45^+^ immunofluorescence staining of livers sections from control and MSC-treated mice demonstrated a massive reduction of leukocyte and macrophage infiltration. *Data* are shown as mean ± standard error of the mean of 10 random high-power fields per mouse. *P < 0.05; **P < 0.01; ***P < 0.001

Mice were sacrificed 24, 48, and 72 h after MSCs intravenous infusion (Additional file 1: Figure S2A). Nine control and one MSC-treated mouse died before sacrifice. Six of the nine remaining control livers were soft and shrunken with extensive extravasated blood, which was gradually reduced at later time points. By contrast, none of the remaining six MSC–treated livers showed gross pathological changes, even 24 h after MSC infusion (Fig. 1a).

Hematoxylin and eosin (HE)-stained liver sections from control mice revealed dramatical hepatocellular death with cytoplasmic vacuolization, panlobular mononuclear CD45-positive leukocyte infiltration (particularly F4/80-positive macrophages), and severe distortion of liver tissue architecture. By contrast, liver sections from MSC–treated mice rarely showed periportal immune cell infiltration with edema and fibrin deposition (Fig. 1d–f; Additional file 1: Figure S2C–E). Also, flow cytometry analysis of total lymphocytes showed that macrophages infiltrated into control livers more than MSC-treated livers (Fig. 1c; Additional file 1: Figure S2B).

During the 7-day follow-up period after cell transplantation, nine of the 18 control mice successively died, with mortality rate reaching 50 %. By contrast, better survival was observed for MSC–treated mice, with only one mouse dying during the observation period (Fig. 1b).

Overall, these results demonstrate that MSC inhibits the development of histopathological changes and immune cell infiltration and reduces mortality among mice with TAA-induced FHF.

### MSC therapy suppresses CCl_4_-induced chronic liver fibrosis and down-regulates infiltrating macrophages

**Fig. 2:**
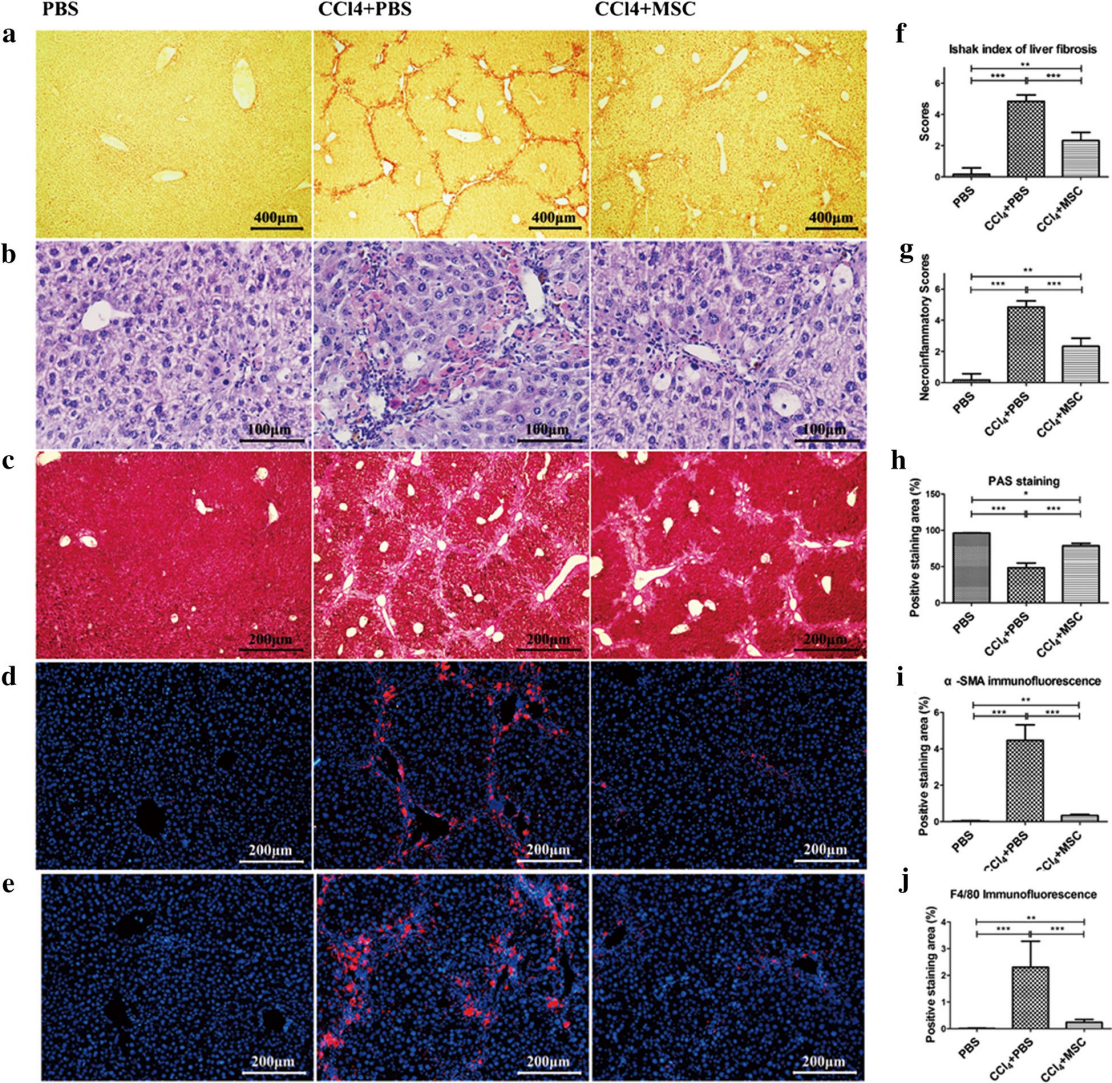
MSC treatment suppresses inflammatory infiltration and down-regulates activated HSCs, inhibiting fiber deposition in CCl_4_-induced chronic liver fibrosis. **a** Sirius Red, **b** HE, and **c** PAS staining of liver sections from control and MSC-treated mice. **d** α-SMA immunofluorescence staining of liver sections shows a down-regulation of activated HSCs. **e** F4/80 immunofluorescence staining of liver sections from normal, control and MSCs-treated mice. **f**, **g** Fibrosis and necroinflammatory scores determined using the scoring system of Ishak [31]. **h**, **i**, **j** Quantification of PAS-positivity, α-SMA-positivity and F4/80-positivety. *Data* are shown as mean ± standard error of the mean of 10 random high-power fields per mouse. *P < 0.05; **P < 0.01; ***P < 0.001

In order to observe changes in liver fibrosis after mice were treated with CCl_4_ and MSC, liver sections (Additional file 1: Figure S3) were stained with Sirius Red to identify collagen deposition. Six mice from normal, control and MSC groups were sacrificed for various microscopic evaluations 3 weeks after MSC infusion (Additional file 1: Figure S4A). Sirius Red-stained liver sections revealed massive collagen deposition in livers from control mice (Fig. 2a, f). MSC treatment also noticeably decreased collagen deposition in mice with TAA-induced chronic liver fibrosis, although this effect was less significant than that observed in mice with CCl_4_-induced chronic liver fibrosis (Additional file 1: Figure S3). The results of immunofluoresence staining of Collagen1 (Col-1) and Collagen3 (Col-3), which are primary contributors to collagen deposition, was consistent with that of Sirius Red staining (Additional file 1: Figure S4B, C, D, E). Also, periodic acid-Schiff (PAS)-staining of liver sections revealed that MSC treatment improved glycogen synthesis and storage (Fig. 2c, h). Moreover, control liver sections around sinus hepaticus were significantly positive for α-smooth muscle actin (α-SMA), a marker of activated HSCs, whereas MSC-treated liver sections showed a sizeable reduction in α-SMA positivity (Fig. 2d, i).

Microscopic evaluation of HE-stained liver sections revealed massive inflammatory infiltration, particularly F4/80-positive macrophages, in livers from control mice. By contrast, MSC treatment markedly down-regulated F4/80-positive macrophage infiltration (Fig. 2b, e, g, j). Taken together, these results demonstrate that MSC therapy suppresses liver fibrosis by down-regulating macrophage infiltration and promoting HSC apoptosis or decreasing the activated HSCs.

### MSCs inhibit hepatocellular apoptosis and enhance liver regeneration in vivo

**Fig. 3:**
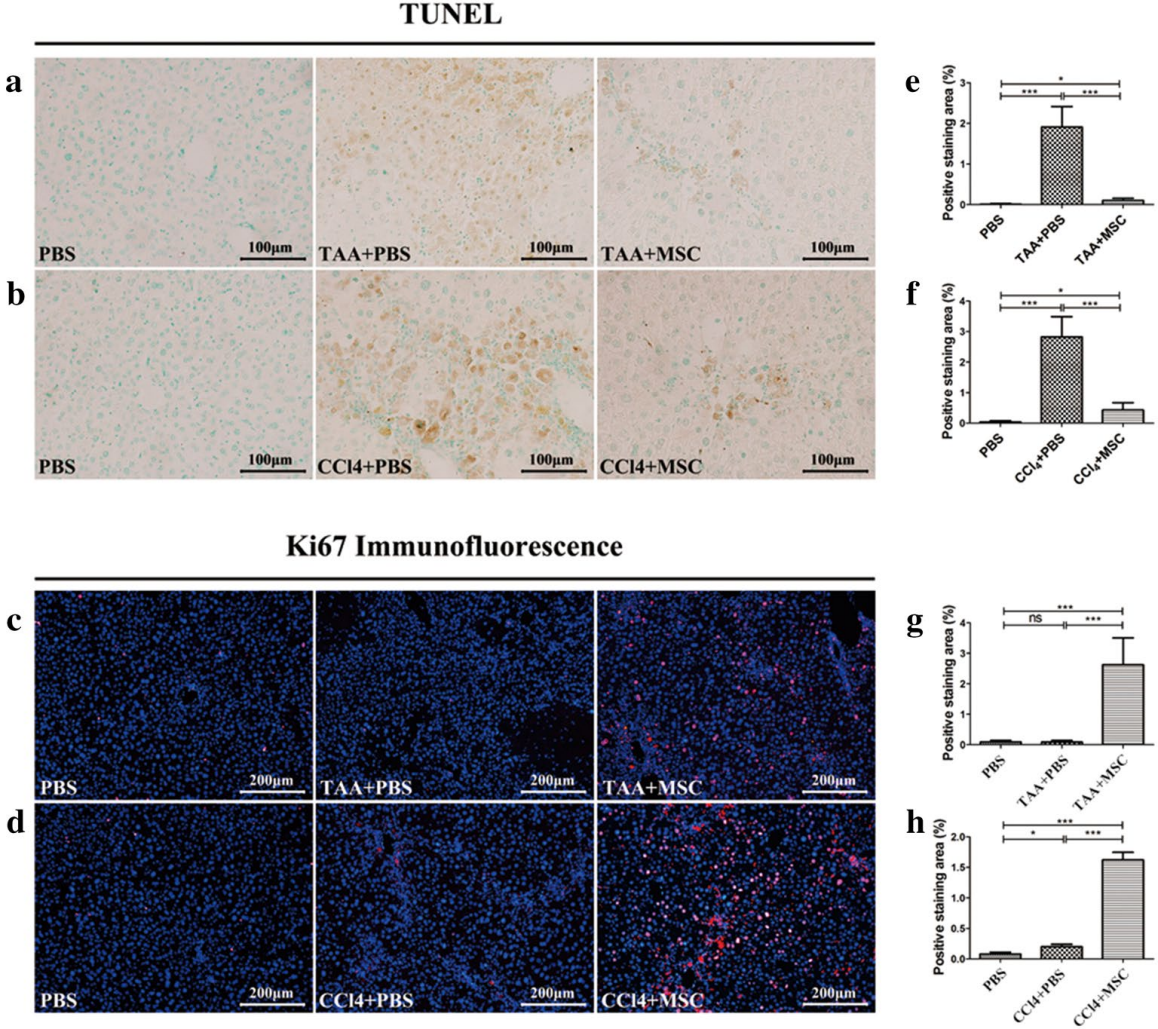
MSC delivery decreases apoptosis and enhances hepatocellular proliferation in mice with liver failure. Liver sections from **a** TAA-stimulated and **b** CCl_4_-stimulated mice were stained by TUNEL (*dark brown* nuclei, large for hepatocytes) and counterstained with methyl green (*light blue*). Ki67 immunofluorescence staining of liver sections from **c** TAA-stimulated and **d** CCl_4_-stimulated mice.Quantification of TUNEL-positivity in **e** TAA-stimulated and **f** CCl_4_-stimulated livers. Quantification of Ki67-positivity in **g** TAA-stimulated and **h** CCl_4_-stimulated livers. *Data* are shown as mean ± standard error of the mean of 10 random high-power fields per mouse. *P < 0.05; **P < 0.01; ***P < 0.001

To determine whether MSCs treatment reduces hepatocellular apoptosis, we examined TUNEL-reactive hepatocyte nuclei in liver sections. In control mice with FHF and chronic liver fibrosis, many large apoptotic hepatocyte nuclei were observed, yet only few such nuclei were observed after MSC treatment. Furthermore, the extravasated blood observed after TAA stimulation disappeared after MSC infusion (Fig. 3a, b). Quantification of these observations confirmed a dramatic reduction in TUNEL-positivity in MSC-treated mice with FHF and chronic liver fibrosis (0.10 ± 0.06 or 0.40 ± 0.05 % per field of view, respectively) compared with control mice (1.92 ± 0.40 or 2.83 ± 0.30 % per field of view) (Fig. 3e, f), demonstrating that MSC effectively inhibits hepatocellular death in models of liver failure.

The therapeutic effects of MSCs may rely on the launch of endogenous repair programs. Hepatocytes positive for the proliferation marker Ki67 were quantified in mice with FHF and chronic liver fibrosis and compared with those in control mice (Fig. 3c, d). Whereas few Ki67-positive hepatocytes were observed in control livers (0.08 ± 0.04 or 0.20 ± 0.03 % per field of view), many were observed in MSC–treated liver (2.62 ± 0.50 or 1.62 ± 0.20 % per field of view) (Fig. 3g, h). These findings demonstrate that MSC treatment inhibits hepatocellular apoptosis and stimulates liver regeneration programs in mice with liver failure.

### MSC-CM partially ameliorates FHF, but dramatically improves chronic liver fibrosis

**Fig. 4:**
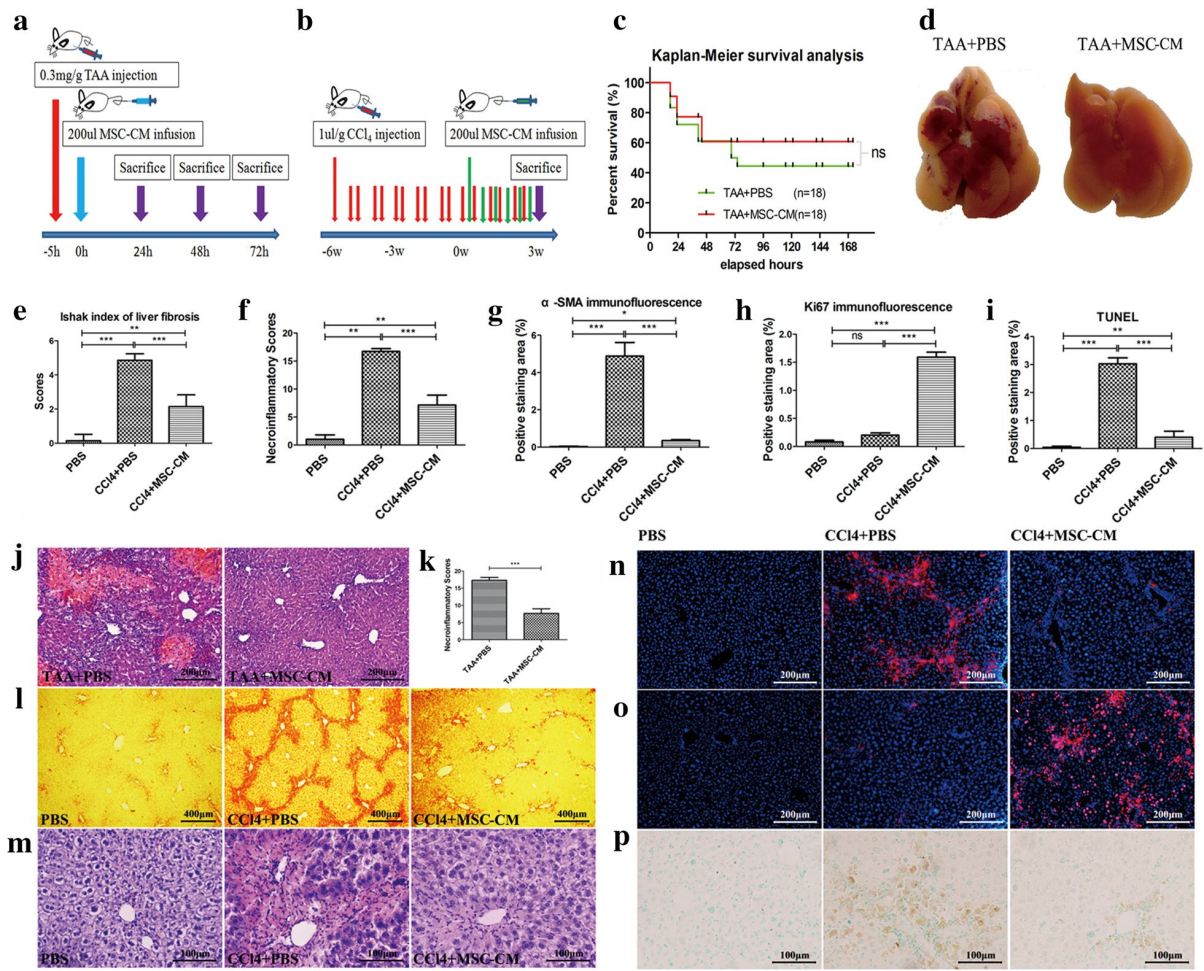
MSC-CM delivery ameliorates TAA-stimulated FHF and CCl_4_-stimulated liver fibrosis. **a** Schema depicting MSC-CM treatment for FHF. **b** Schema depicting MSC-CM treatment for chronic liver fibrosis. **c** Kaplan–Meier survival analysis of mice with TAA-induced FHF. **d** MSC-CM treatment substantially improved the gross histopathological appearance of TAA-stimulated livers. **e**, **f** Fibrosis and necroinflammatory scores were determined using the scoring system of Ishak for CCl_4_-stimulated livers. Quantification of (**g**) α-SMA-, (**h**) Ki67-, and (**i**) TUNEL-positivity. **j** HE staining of liver sections from control and MSC-CM-treated TAA-stimulated livers. **k** Necroinflammatory scores determined by Necroinflammatory score system of Ishak in TAA-stimulated livers. **l** Sirius red, **m** HE, **n** α-SMA immunofluorescence, **o** Ki67 immunofluorecence, **p** TUNEL staining of liver sections from CCl_4_-stimulated all group. *Data* are shown as mean ± standard error of the mean of 10 random high-power fields per mouse.*P < 0.05; **P < 0.01; ***P < 0.001

MSCs naturally support hematopoiesis by secreting several trophic molecules, including soluble extracellular matrix glycoproteins, chemokines, cytokines, and growth factors. To determine whether MSC-CM plays an important role in improving liver failure, MSC-CM was intravenously infused into mice with FHF or chronic liver fibrosis. Interestingly, the therapeutic effect of MSC-CM infusion was similar to that of MSC infusion, although there were notable differences in their courses of action for FHF. Mice with FHF were sacrificed 24, 48, or 72 h after MSC-CM infusion (Fig. 4a). Six of the eight survival control livers were soft and shrunken with extensive extravasated blood. Microscopic evaluation of HE-stained control liver sections consistently revealed massive hepatocellular death with cytoplasmic vacuolization, hemorrhage and inflammatory infiltration. These severe pathological changes were also observed in MSC-CM-treated livers 24 and 48 h after infusion, although a therapeutic effect was observed 72 h after MSC-CM infusion (Fig. 4d, j, k). However, MSC-CM treatment did not significantly improve survival rate (55.6 % for MSC-CM-treated vs. 44.5 % for control group) (Fig. 4c). Therefore, it is likely that MSC-CM enhances the liver repair system only at later stages of self-recovery.

To access recovery from chronic liver fibrosis, six of normal, control and MSC-CM groups were sacrificed for various microscopic evaluations 3 weeks after MSC-CM infusion (Fig. 4b). Sirius Red-staining, HE-staining and α-SMA immunofluoresence-staining of liver sections revealed that MSC-CM treatment suppressed collagen fiber deposition, inhibited inflammatory infiltration, and promoted activated HSC apoptosis (Fig. 4e–g, l–n). Also, MSC-CM-treated livers exhibited less TUNEL-positivity and more Ki67-positive hepatocyte nuclei, indicating that MSC-CM trophic molecules inhibit hepatocyte apoptosis and enhance liver regeneration (Fig. 4h, i, o, p). Overall, these results show that MSC-CM improves chronic liver fibrosis, but only partially improves FHF.

### MSCs promote macrophage line RAW264.7 apoptosis and MSC-CM promotes apoptosis and inhibits proliferation of HSC line LX-2

**Fig. 5:**
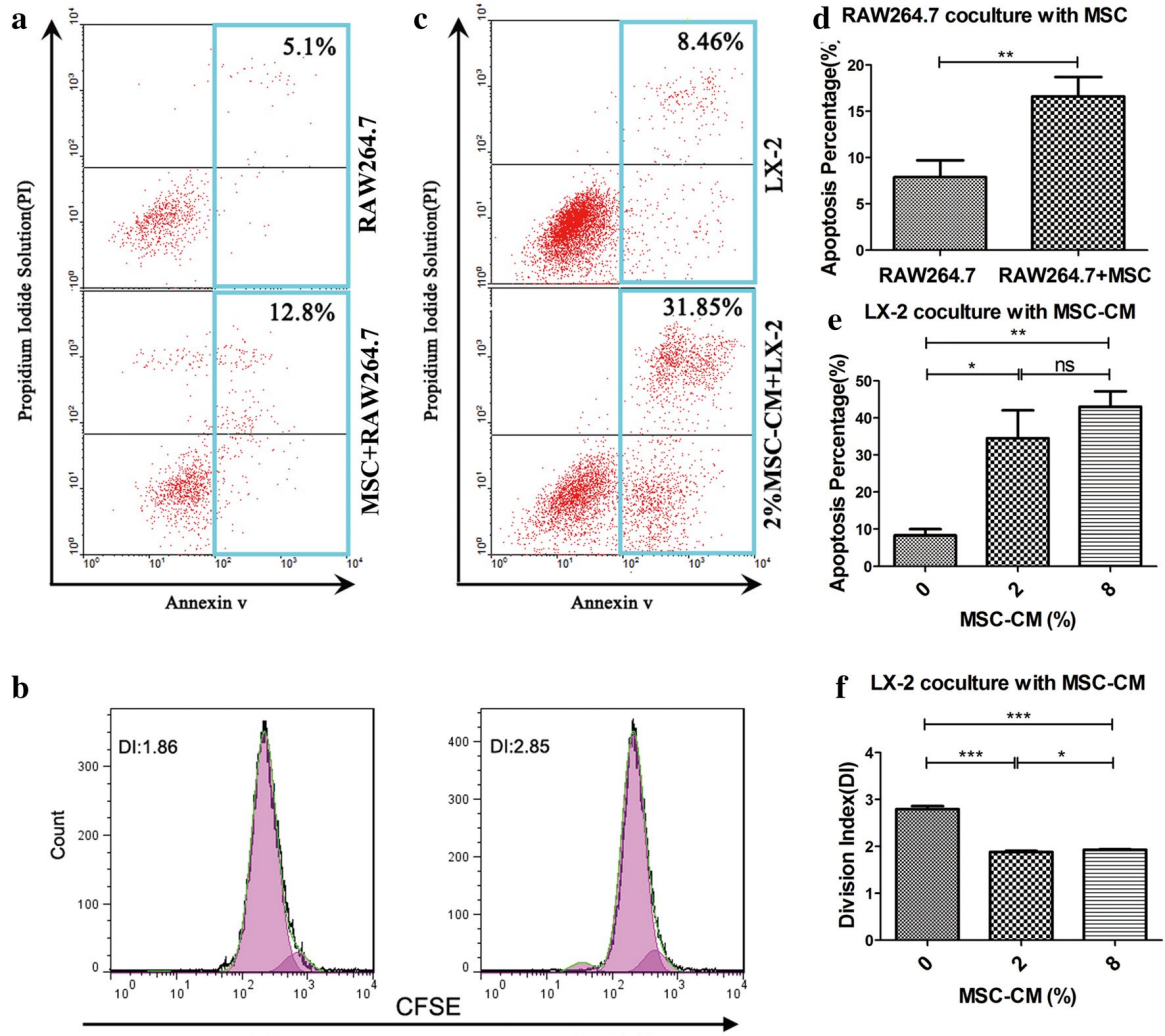
MSCs promote RAW264.7 apoptosis, and low concentration of MSC-CM facilitates LX-2 apoptosis and inhibition of proliferation. **a** Co-culture of RAW264.7 and MSCs induced massive apoptosis of RAW264.7 48 h later, shown using a fluorescent live/dead assay. **b** A low concentration of MSC-CM suppressed LX-2 proliferation 48 h later. **c** A low concentration of MSC-CM increased LX-2 apoptosis 48 h later, shown using a fluorescent live/dead assay. **d** Percentage of apoptosis for co-culture and RAW264.7-alone conditions. **e** Percentage of apoptosis for co-culture and LX-2-alone conditions. **f** Division index analysis of co-culture and LX-2-alone conditions. Experiments were performed in triplicate. *Data* are shown as mean ± standard deviation. *P < 0.05; **P < 0.01; ***P < 0.001

Macrophages and HSCs have important role in the pathogenesis of FHF and chronic liver fibrosis, respectively. To determine whether MSCs down-regulate macrophages and HSCs apoptosis in vivo via a direct effect of MSCs themselves or an indirect effect of MSC-secreted soluble factors, we examined the effect of MSCs or MSC-CM on in vitro apoptosis and proliferation of RAW264.7 or LX-2. After co-culture of RAW264.7 and MSCs for 48 h, a two-fold increase in apoptosis was observed (16.6 ± 2.0 % co-culture group vs. 7.9 ± 1.2 % control group) (Fig. 5a, d), whereas MSCs and MSC-CM did not effect on the proliferation of RAW264.7, MSC-CM did not promote RAW264.7 apoptosis.

After LX-2 was supplemented with 2 % MSC-CM in co-culture for 48 h, we observed a massive apoptosis of LX-2 (34.5 ± 5.0 in 2 % MSC-CM-treated group vs. 8.3 ± 1.5 % control group). With 8 % MSC-CM, no significant increase in LX-2 apoptosis was observed (Fig. 5c, e). Supplementation with 2 % MSC-CM also suppressed the proliferation of LX-2 (1.88 ± 0.02 in 2 % MSC-CM group vs. 2.80 ± 0.06 % control group) (Fig. 5b, f). Taken together, these results suggest that MSCs themselves directly facilitate macrophage apoptosis, whereas HSC apoptosis and inhibition of proliferation occur via MSC-CM.

### MSCs and MSC-CM have anti-inflammatory effects on TAA- and CCl_4_-stimulated splenocytes, respectively

**Fig. 6:**
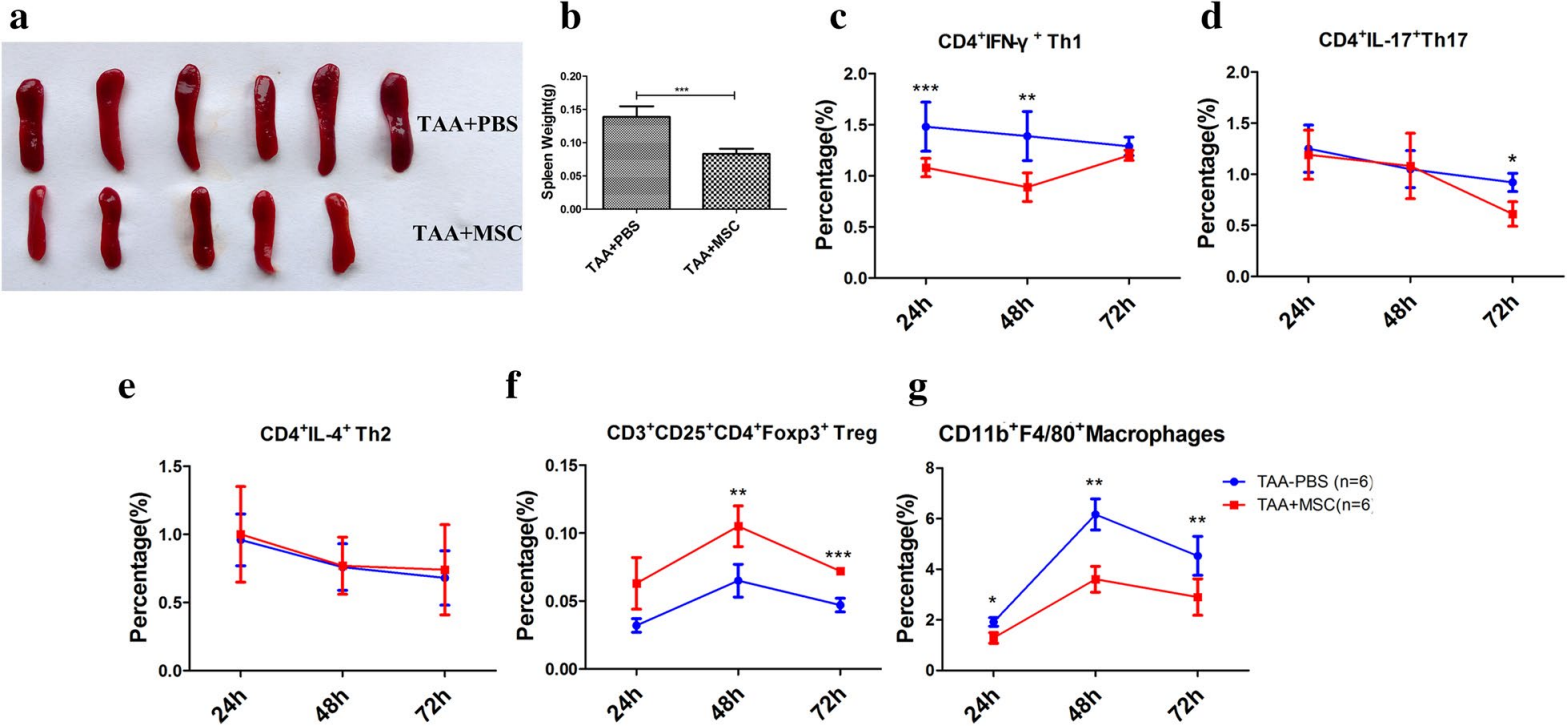
MSC and MSC-CM down-regulate macrophages and converts the CD4^+^ T lymphocyte system into an anti-inflammatory state in TAA-stimulated spleens. **a** Spleens from control and MSC-treated TAA-stimulated mice 72 h after MSC treatment. **b** Spleen weight in MSC-treated mice with FHF. Quantification of (**c**) Th1, (**d**) Th17, (**e**) Th2, (**f**) Treg and (**g**) macrophages in spleen

We examined the subset distribution of CD4^+^ T lymphocytes in spleens from control, MSC-treated, and MSC-CM-treated mice using flow cytometry. Based on our prior experience, we expected that the subset distribution of CD4^+^ T lymphocytes in spleens would not be altered by MSC-CM treatment of TAA-stimulate mice. However, MSC infusion down-regulated pro-inflammatory Type 1 T helper (Th1) and Th17 cells (Fig. 6c, d; Additional file 1: Figure S5A, B) and up-regulated anti-inflammatory regulatory T (Treg) cells in mice with FHF (Fig. 6f; Additional file 1: Figure S5D), whereas the distribution of anti-inflammatory Th2 cells- was not significantly changed (Fig. 6e; Additional file 1: Figure S5C). Consistently, the size of spleen in mice from MSC-CM treatment group was smaller than control group mice, which indicates that mice from MSC-CM treatment group were under lower inflammatory state compared with control group (Fig. 6a, b). Therefore, MSCs directly exert immunosuppressive effects in mice with TAA-induced FHF.

**Fig. 7:**
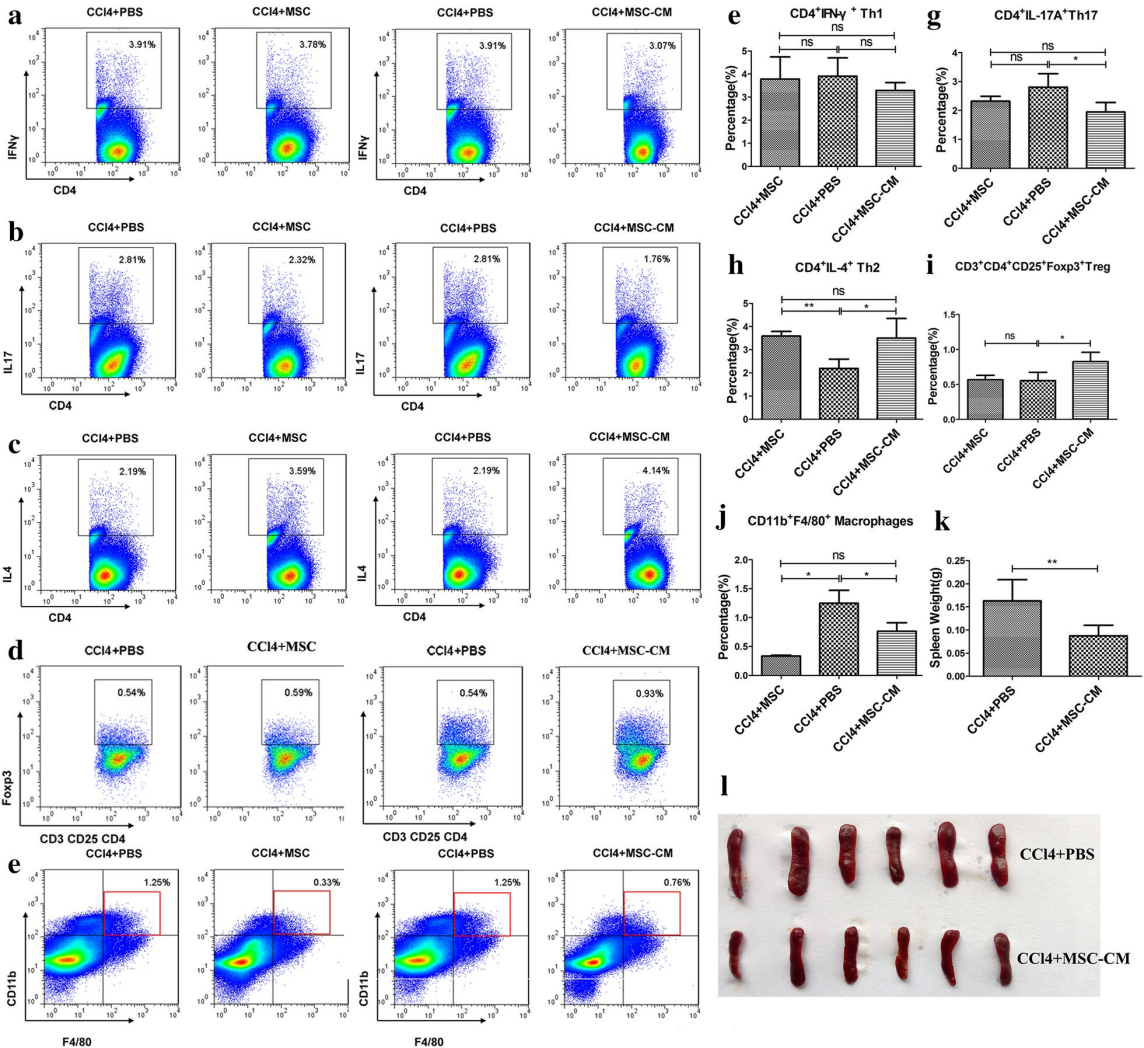
MSC and MSC-CM down-regulate macrophages and converts the CD4^+^ T lymphocyte system into an anti-inflammatory state in CCl_4_-stimulated spleens. Both MSC and MSC-CM treatment had no significant effect on (**a**) Th1. A significant reduction of (**b**) Th17 was observed 3 weeks after MSC-CM but not MSC treatment. However, (**c**) an increase in Th2 and (**e**) a reduction in macrophages was observed after both MSC and MSC-CM treatment. MSC-CM but not MSC treatment up-regulated (**d**) Treg. Quantification of (**f**) Th1, (**g**) Th17, (**h**) Th2, (**i**) Treg, and (**j**) macrophages in the spleen. **k** Spleen weight in MSC-CM-treated mice with chronic liver fibrosis. **l** Spleens from control and MSC-CM-treated CCl_4_-stimulated mice

By contrast, both MSC and MSC-CM treatment exerted immunosuppressive effects in CCl_4_-induced chronic liver fibrosis, although better therapeutic effects were observed after MSC-CM delivery. MSC-CM increased levels of Th2 and Treg cells (Fig. 7c, d, h, i), and reduced levels of Th17 cells (Fig. 7b, g), whereas levels of Th1 cells were unchanged (Fig. 6o, j). Moreover, the size of spleen from MSC-CM treatment mice was smaller than control mice (Fig. 7k, l). Comparatively, MSC treatment did not affect Th17 and Treg cells and only slightly alters inflammatory state in mice with chronic liver fibrosis. Also, MSC and MSC-CM treatment substantially down-regulated macrophages in the spleen of mice with acute and chronic liver failure (Figs. 6g, 7e, j; Additional file 1: Figure S5E), consistent with effects observed in the liver. Therefore, in mice with CCl_4_-induced chronic liver fibrosis, immunosuppressive effects are mainly attributed to MSC-CM. There results demonstrate that MSCs themselves exert immunosuppressive effects in mice with TAA-induced FHF, whereas MSC-CM underlies the immunosuppressive effects in mice with CCl_4_-induced chronic liver fibrosis.

## Discussion

**Fig. 8:**
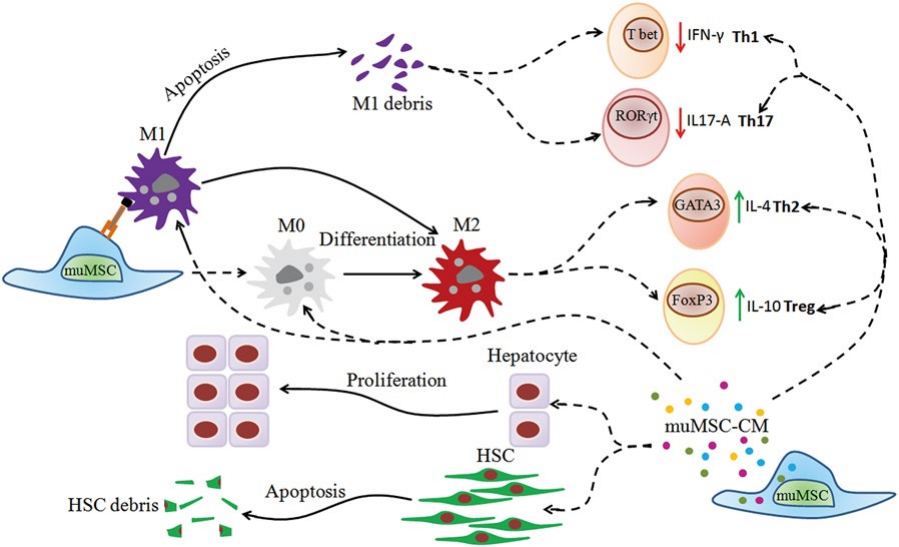
Schema depicting mechanisms of MSC and MSC-CM therapy. MSCs and MSC-CM containing MSC-secreted soluble factors ameliorate TAA-induced acute liver injury and CCl4-induced chronic liver fibrosis by acting on hepatocytes, macrophages, CD4 + helper T lymphocytes, and HSCs, which have important roles during the pathogenesis of liver injury. muMSC: murine mesenchymal stem cell; M0: undifferentiated macrophage; M1: M1 macrophage; M2: M2 macrophage

Several clinical and animal model-based trials show promising and desirable therapeutic effects of MSCs in ameliorating FHF, chronic liver fibrosis, and even cirrhosis [15]. Recent investigations focusing on MSC-CM reveal several therapeutic mechanisms of MSCs treatment [16]. However, which mechanism of action has a leading role in improving FHF or chronic liver fibrosis is inconclusive, even though this information is important for future researches. In the present study, we systematically investigated the effects of MSCs and MSC-CM treatment in mouse models of TAA-induced FHF and CCl_4_-induced chronic liver fibrosis in terms of enhancing liver regeneration, reducing hepatocellular apoptosis, down-regulating macrophage infiltration, altering the CD4^+^ T system into an anti-inflammatory state and promoting HSC apoptosis and inhibition of proliferation (Fig. 8).

We found that MSC and MSC-CM infusion similarly stimulated liver regeneration and suppressed hepatocelluar death in mice with acute and chronic liver failure. These results are consistent with a previous report that low-concentrations MSC-CM are sufficient to promote hepatocelluar proliferation and inhibition of hepatocyte apoptosis [14]. Higher concentrations of MSC-CM, however, did not confer better therapeutic effect, most likely because MSC-CM includes small amounts of inhibitory components, such as TNF-α and TGF-β, whose negative effects at higher concentrations offset the therapeutic effects of trophic factors.

Another characteristic in common between MSC and MSC-CM therapy is the substantial reduction of macrophages in the liver and spleen. Several studies show that distinct mechanisms of MSCs are responsible for the down-regulation of pro-inflammatory macrophages. For example, MSCs mediate a switch from pro-inflammatory M1-type macrophages to anti-inflammatory M2-type macrophages [17, 18]. Also, another study shows that MSC-CM suppresses M1-type macrophages in mice with endotoxin-induced acute lung injury [19]. However, co-culture of MSC-CM with RAW264.7 did not induce apoptosis of RAW264.7 in the present study, which could be partly attributed to functional differences among MSC populations that possess different spectrums of secreted factors [20].

Immunosuppression is an important therapeutic mechanism of MSCs in various models of autoimmune disease [21], and this is also true of MSC-CM [22, 23]. The present study further demonstrates the immunosuppressive effect of MSC treatment, converting the body into an anti-inflammatory state by up-regulating anti-inflammatory Treg cells and reducing pro-inflammatory Th1 and Th17 cells in TAA-induced FHF and CCl_4_-induced chronic liver fibrosis (Figs. 6, 7), which could partially explain the beneficial effects of MSC. MSC-CM delivery also led to an immunosuppressive response in chronic liver fibrosis, although immunosuppressive effects of MSC-CM treatment were not observed in mice with TAA-induced FHF. Therefore, we infer that MSCs themselves exert immunosuppressive effects in TAA-induced FHF, whereas MSC-secreted soluble factors dominate the immunosuppressive effects of MSC treatment in CCl_4_-induced chronic liver fibrosis. Moreover, the establishment of an anti-inflammatory state after MSC or MSC-CM treatment could occur indirectly via the up-regulation of M2-type macrophages, which secrete various anti-inflammatory factors such as CCL-1 and IL-10 that up-regulate Th2 and Treg cells [24].

The principal mediators of hepatic fibrosis are HSCs, which substantially proliferate and produce various extracellular matrices during the pathogenesis of liver fibrosis. Both MSC and MSC-CM treatment dramatically reduced activated HSCs-myofibroblasts. The down-regulation of activated HSCs may be achieved by different pathways. For example, fibrous scar-produced myofibroblasts can revert into inactive phenotypes [25]. Alternatively, the massive apoptosis and inhibition of proliferation of LX-2 that we observed during co-culture of MSC-CM and LX-2 may be explained by a previously reported immunomodulatory mechanism [26]. However, the therapeutic effect of MSCs treatment has been called into question [27], with recent studies showing that MSC infusion can accelerate the progress of fibrosis via the conversion of MSCs into fibrous scar-produced myofibroblasts [28–30]. Therefore, the therapeutic effect of MSC infusion may be mainly realized by the actions of MSC-secreted factors that mediate the massive apoptosis of HSCs based on our results in vivo and LX-2 coculture with MSC-CM in vitro, thereby counteracting the negative effects of MSC-derived myofibroblasts.

Both MSC and MSC-CM therapy improved TAA-induced FHF and CCl_4_-induced chronic fibrosis by acting on hepatocytes, macrophages, CD4^+^ T lymphocytes, and HSCs. However, the primary therapeutic mechanisms of MSC therapy differ between these two models of liver failure. MSCs can achieve healing of injury by two different modes—direct interaction with various target cells or secretion of various soluble molecules. For TAA-stimulated FHF, MSC but not MSC-CM significantly ameliorated injury by promoting hepatocelluar proliferation and inhibiting hepatocyte apoptosis, suppressing macrophage infiltration, and converting the CD4^+^ T lymphocyte system into an anti-inflammatory state. MSC-CM also stimulated liver regeneration and inflammatory infiltration 72 h after delivery. However, because the death of TAA-stimulated mice generally occurred during the first 48 h after TAA injection, MSC-CM infusion did not decrease mortality rates during this early time period (44.4 % in the MSC-CM-treated group vs. 55.5 %in the control group). Therefore, MSCs themselves play the predominant therapeutic role in MSC therapy for FHF. Several previous studies demonstrate extensive interactions between MSCs and various immune cells such as activated T cells. Immunosuppressive outcomes from the interplay between MSCs and various immune cells may be the main mechanism by which MSC infusion prevents the death of mice during the first 48 h after TAA injection. By contrast, for CCl_4_-stimulated chronic liver fibrosis, MSC-CM plays a predominant therapeutic role by enhancing the liver repair system, inhibiting inflammatory infiltration, and promoting the apoptosis of HSCs, which outweighs the negative effects of MSC-derived myofibroblasts. Therefore, MSC-secreted soluble factors are the primary route of action during MSC therapy for CCl_4_-induced fibrosis.

## Conclusion

In conclusion, we found that both MSCs and MSC-CM induce integrated therapeutic effects on mice with liver failure, although the two treatments differ in their dominant therapeutic modes. In term of MSCs therapy, our findings provide the clinical doctor with advisable remedies that MSC treatment is more suitable for FHF, whereas delivery of MSC-CM is more suitable for chronic liver fibrosis.

## Additional file

10.1186/s12967-016-0792-1 Supplemental materials, methods and results.
