# Supplementary material for: Mesenchymal stem cells and their secreted molecules predominantly ameliorate fulminant hepatic failure and chronic liver fibrosis in mice respectively
Source: J Transl Med. 2016 Feb 9;14:45. doi: 10.1186/s12967-016-0792-1 (PMC4746907; doi:10.1186/s12967-016-0792-1)
Supplement: Supplementary file 1 — 10.1186/s12967-016-0792-1 Supplemental materials, methods and results. [file 12967_2016_792_MOESM1_ESM.docx]

**Mesenchymal stem cells and their secreted molecules predominantly ameliorate fulminant hepaticfailure and chronic liver fibrosis in mice respectively**

Biao Huang^a,†^, Xixi Cheng^a,†^, Dan Wang^a,†^, Wenjing Huang^a^, Zha la Gahu^b^, Huafeng Wang^a^, Huan Zhang^a^, Zhenyi Xue^a^, Yurong Da^a^, Ning Zhang^a^, Yongcheng Hu^c^, Zhi Yao^a^, Liang Qiao^d^, Fei Gao^e,^* and Rongxin Zhang^a,^*

**Supplemental Data:**

**Materials and Methods:**

**Isolation and Culture of bone-derived MSCs**

The femurs and tibiae were collected from 2- to 3-week-old female ICR mice. The epiphyses were removed, bonemarrow was flushed out with PBS, and the bone cavities were washedthoroughly with α-modified minimal essential medium (α-MEM) by drawing and expelling with a syringe. The compactbones were excised into chips of about 1 mm^3^ into plasticculture dishes, suspended inα-MEM containing 10% fetal bovine serum (FBS, GIBCO) fromselected lots in the presence of 1 mg/ml of collagenase II (GIBCO), andincubated for 2 hours at 37°C with shaking at a speed of 200rpm. The released cells were removed, and the bone fragmentswere washed three times with PBS and one time with α-MEM, followed by incubationinα-MEM containing 10% selected FBS at 37°C in 5% CO2.Medium was changed every 4-5 days. The adherent cells(passage 0) wereharvested by 0.25% trypsin-EDTA (GIBCO) digestion and passaged on7-8 day of incubation. The cells at the fifth passagewere used for the following experiments.Also, the released cells after digestion were propagatedseparately in the same culture medium in some experiments.MSCs were characterizedby their fibroblast-like morphology, a distinctive panel of surface markers (CD29+, CD44+, CD45-, CD86-, CD135- and CD11b-) and theirdifferentiation potential towards adipocytes and osteoblasts in special induction medium. FACS staining and lineagedifferentiation induction were performed as describedpreviously.

**Preparation of MSC-CM**

For the generation of MSC-CM, cells were allowed to grow to 80% to 90% confluence (approximately 3×10^6^MSCs per 55-cm^2^ dish), washed thoroughly, and cultured in 10 mL serum-freeα-MEM. Conditioned medium was collected 24 hours later. Conditioned growth medium was concentrated 25-fold through ultrafiltration units (Millipore, Bedford, MA) with a 3-kDa cutoff. Protein concentrations were estimated using the BCA Protein Assay Kit, and 0.5 mg protein (about 200μl MSC-CM) was used for in vivo studies. α-MEMmixed at a 50:1 ratio with the 25-fold concentrated MSC-CM (2% MSC-CM), and at a 12.5:1ratio for 8% MSC-CM.2% and 8% MSC-CM was used for in vitro co-culture studies.

**Animal Model and treatment protocols**

Six-to-eight-week-old female ICR mice weighing 20-25g were used for the construction of animal model.

**Induction of FHF**

FHF was induced by a single dose of thioacetamide (TAA; dissolved into 40μg/μl with sterile PBS) injected intraperitoneally. Preliminary experiments were performed to examine the effect of TAA on mortality rate at doses of 0.2, 0.3 and 0.4 mg/g. It was found that TAA-induced mortality rate reach 50% at a dose of 0.3mg/g; thus, this dose was selected for use in the present study. Mice in the control group received sterile PBS only[^1^](#_ENREF_1). After 5 hours of TAA injection, 200μl MSC-CM or 1×106 MSCs and 200μl PBS solution (vehicle control) was infused into the tail vein. We used 6 animals per group for tissue collection after sacrifice at 24, 48, 72 hours after treatment and 6 animals per group for survival analysis.TAA-induced FHF is accompanied by characteristic changes in the gross appearance of the liver[^2^](#_ENREF_2)^,^[^3^](#_ENREF_3).

**Induction of Chronic Liver fibrosis**

To induce liver fibrosis, mice received 12 consecutive intraperitoneal injections (1 μl/g body weight) of CCl4: olive oil (1:1) twice per week for 6 weeks. Injection of olive oil alone served as a control. 1×106 MSCs were infused into the tail vein, or 200 μl MSC-CM were infused into tail vein twice per week for 3 consecutive weeks at the sixth week of CCl4 injections. Control groups mice receive the same volume PBS[^4^](#_ENREF_4). We used 8 animals per group for tissue collection after sacrifice at 3weeks after treatment. CCl4: olive oil (1:1) was persistently injected during treatment of MSCs and MSC-CM.CCl4-induced chronic liver fibrosis is typically associated with extensive collagen deposition and activation of HSCs[^5^](#_ENREF_5).In addition, Liver failure often leads to an alteration of immune balance, converting the body into a pro-inflammatory state[^6^](#_ENREF_6).

**Immunohistochemistry**

Eight-micrometer-thick sectionsof formalin-fixed tissue were deparaffinized, rehydrated,and blocked in 3% hydrogen peroxide in ethanolfor 15 minutes after baking at 60°C for 1 hour.For CD45-FITC, F4/80-APC, F4/80, Col-1, Col-3, α-SMA and Ki67 immunohistochemistry,sections were treated in 10 mM citratebuffer at pH 6.0 using a digital pressure cooker, blockedwith 1.5% bovine serum albumin (BSA) for 30 minutes, and incubatedwith CD45-FITC (eBioscience), F4/80-APC (eBioscience), rabbit anti-F4/80(AbDSerotec), rabbit anti-Col-1(abcam), rabbit anti-Col-3 (abcam), rabbit anti-α-SMA (abcam) or rabbit anti-Ki67 (abcam) at a1:200 - 1:300 dilution overnight at 4℃, respectively. Sections for CD45-FITC, F4/80-APC, Col-1, Col-3, α-SMA, Ki67 analysis were washed, then Col-1, Col-3, α-SMA, Ki67 was incubated with a donkey anti-rabbit immunoglobulin Gsecondary antibody for 30 minutes at room temperature.Finally, the sections were washed and mounted with medium containing 4′,6-diamino-2-phenyl indole (DAPI, GIBCO). With reference to sections for F4/80 analysis, the sections were washed with PBS three times and incubated with the appropriate secondary antibodies at 37℃for 1 hour.Afterreaction with the DAB chromogen, the sections wererinsed with distilled water, counterstained with hematoxylin.For terminaldeoxynucleotidyltransferase-mediated nick-end labeling (TUNEL) staining, we used the apoTACS-DAB In Situ Apoptosis Detection Kit (Trevigen; Gaithersburg, MD) according to the vendor’sinstructions. The sections were developed using 3,3′-diaminobenzidineand counterstained with hematoxylin.

**Co-culture ofMacrophage line RAW264.7 and MSCs**

MSCs and RAW264.7 were suspended in DMEN (GIBCO) medium supplemented with 10% heat-inactivated FBS, 100 IU/ml penicillin and 100 mg/ml streptomycin. MSCs were plated in 6-well flat-bottom plates at 5×10^4^ cells. Once cells reached confluence, 3×10^6^ RAW264.7 was added to each well (MSC: RAW264.7 ratio ~ 1:6). Controls include RAW264.7 and MSCs cultured alone.

Flow cytometric analysis of experimental groups was performed after 48h of co-culture. Detached cells were stained with APC conjugated anti-mouse F4/80. Apoptosis of RAW264.7 was determined after coculture by co-staining with APC-F4/80 (eBioscience) and FITC conjugated anti-Annexin V. Dead cells were excluded from analysis using propidium iodide (PI). At least 30,000 events were used for analysis[^7^](#_ENREF_7).

**Co-culture ofHuman** **hepatic stellate cell line LX-2 and MSC-CM**

LX-2 were plated in 12-well flat-bottom plates at 2×10^4^ cells with DMEM medium containing 10% heat-inactivated FBS, 100 IU/ml penicillin and 100 mg/ml streptomycin. Once cells reached confluence, DMEM medium was replaced with or without MSC-CM at 2% or 8% for 48h.

Proliferation of LX-2 was assessed using CFSE staining.LX-2 were stained with CFSE (Molecular Probes, Eugene, Oregon, USA) prior to plating. Cells were suspended in 1 ml of PBS and 1 μm of prepared CFSE solution (2.5 μM) and incubated at 37℃ for 15 min with gentle agitation. The labeling reaction was stopped by adding 1 ml of heat-inactivated FBS. Cells were washed in PBS and plated as described.

Flow cytometric analysis of experimental groups was performed after 48h of co-culture. Apoptosis of LX-2 was determined after co-culture by the Annexin-V/propidium iodide (PI) assay. At least 30,000 events were used for analysis.

**Results**

**Phenotype and multipotentstem cell characteristics of infused MSCs.**Phenotypic analysis of MSCsbased onflow cytometry showed that MSCs from passages 5 and 7 were positive for CD29 (95.98% and 99.88%,respectively) and CD44 (98.24% and 98.73%) but negative for CD45 (0.93% and 0.02%), CD31 (0.17% and 0.18%), CD11b (0.64% and 0.09%), CD86 (0.57% and 0.24%) and CD135 (0.06% and 0.06%), and CD31 (0.17% and 0.18%) **(Supplementary Fig.1A)**. These cells exhibitedclassic fibroblast-like morphology**(Supplementary Fig.1B)**.Multipotent stem cell characteristicswere demonstrated via culture in osteogenic or adipogenicdifferentiation conditions.Osteogenic differentiatedMSCs were stainedwith Alizarin Red S on day 21 to identify calcium depositions characteristic of osteoblasts **(Supplementary Fig.1C)**. Adipogenic-differentiatedMSCswere stained with Oil Red O on day 28 to show the existence of lipid droplets **(Supplementary Fig.1D)**. These results indicate that the MSCs used for infusionshowed classic MSC phenotypes and multipotent stem cell characteristics.

**Supplementary Figure Legends and Figures:**

**Supplementary Figure 1.** The morphology of MSCs and MSCs differentiated intoosteocytes, adipocytes and flow cytometry analysis of isolated MSCs phenotype. **(A)** At passage 5-7, cells were harvested and stained for the indicated surface markers as described in Materials and Methods.**(B)**MSCs presented a fibroblast-like morphology (magnification×20). **(C)** Differentiated osteocytes showed calcium deposition by Alizarin Red S staining (magnification×10). **(D)** Differentiated adipocytes revealed intracellular lipid accumulation by oil red O staining (magnification×20).


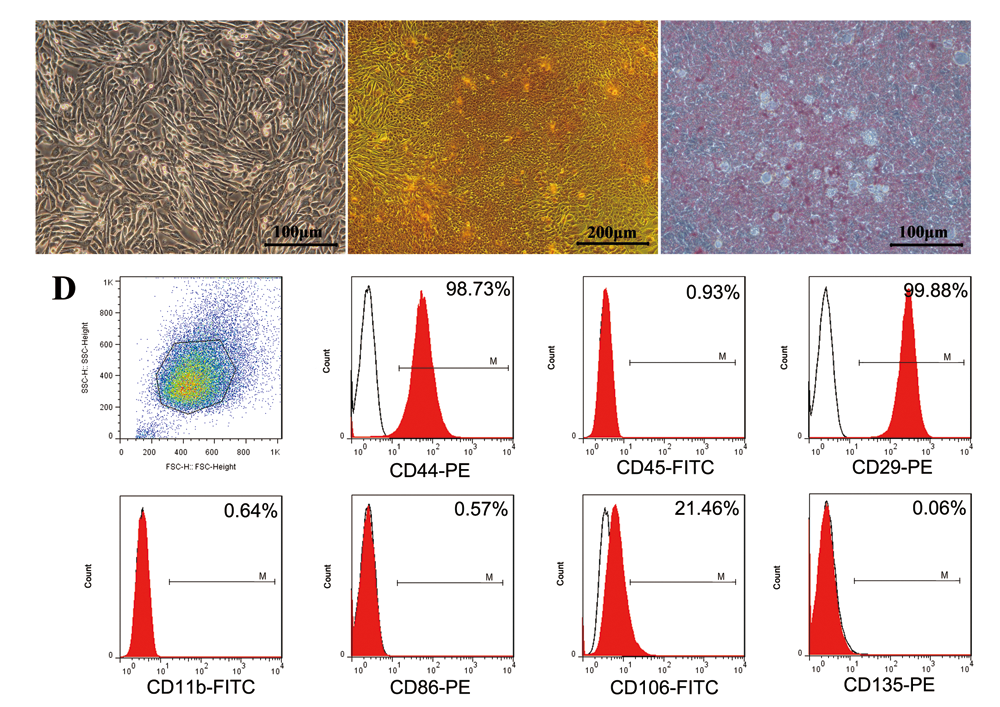


**Supplementary Figure 2.**MSC infusion promotes survival, improves gross and microscopic histopathology, and inhibits the infiltration of leukocytes and macrophages in TAA-induced acute liver injury. TAA-stimulated mice were sacrificed 48h after MSC infusion.**(A)** Schema of MSCs-treated acute liver injury transplants procedure. 1×10^6^MSCs were infused into ICR mice via the tail vein. All groups were sacrificed at indicated time points for sample collection. Zero hour represents the time point of MSCs transplants.**(B)** Necroinflammatory scores determined by Necroinflammatory score system of Ishak. Quantification of **(C)** F4/80-positive and **(D)** CD45-positive staining area.**(E)** Percentage analysis of CD11b^+^F4/80^+^macrophages in control and MSCs-treated livers.Data are shown as mean ± standard error of the mean of 10 random high-power fields per mouse. *P<0.05; **P<0.01; ***P<0.001.


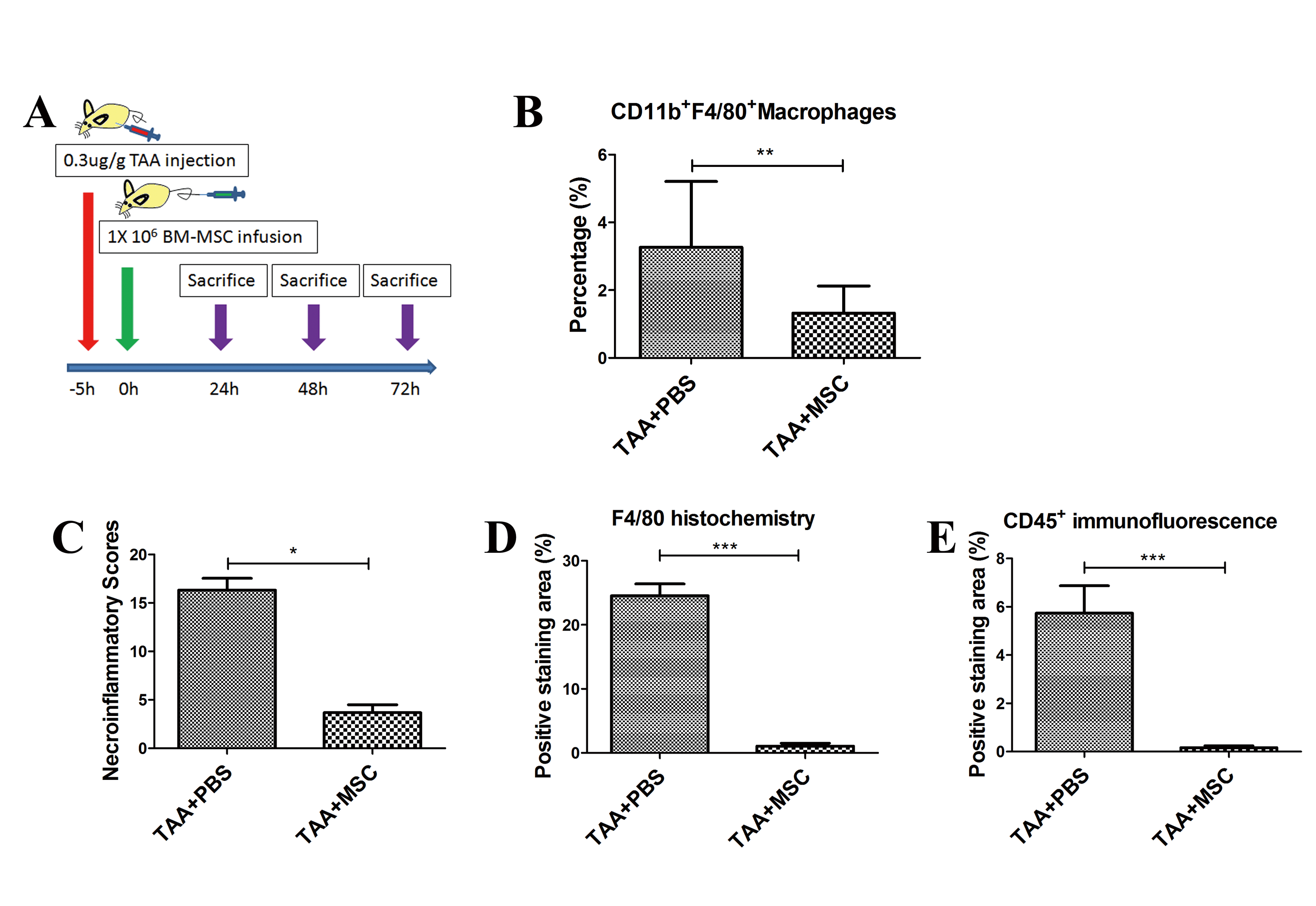


**Supplementary Figure 3.**MSC transplants induced the notable decrease of collagen deposition in the model of TAA-induced fibrosis.


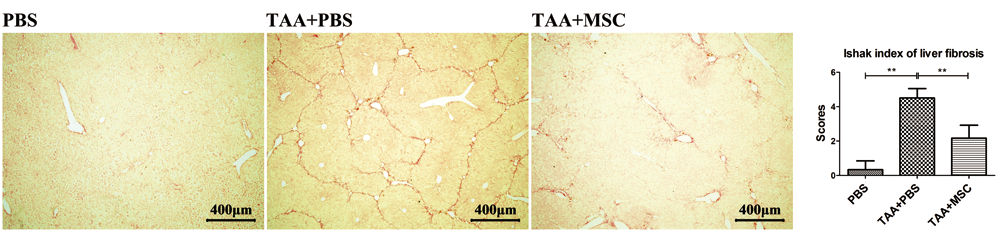


**Supplementary Figure 4.**MSC treatment suppresses inflammatory infiltration and down-regulates activated HSCs, inhibiting fiber deposition in CCl_4_-induced chronic liver fibrosis. CCl_4_-stimulated mice were sacrificed 3 weeks after MSC infusion.**(A)**Schema of MSCs-treated chronic liver fibrosis transplants procedure. 1×10^6^MSCs were infused into ICR mice via the tail vein. All groups were sacrificed at 3 week after MSCs treatment for sample collection.Zero hour represents the time point of MSCs transplants.**(B)** Col-1, **(C)** Col-3immunofluorescence staining of liver sections from all group livers reveals the massive reduction of fiber deposition.Quantification of **(D)** Col-1-positve, **(E)** Col-3-positve staining area.Data are shown asmean±standard error of the mean of 10 random high-power fields per mouse. *P<0.05; **P<0.01; ***P<0.001.


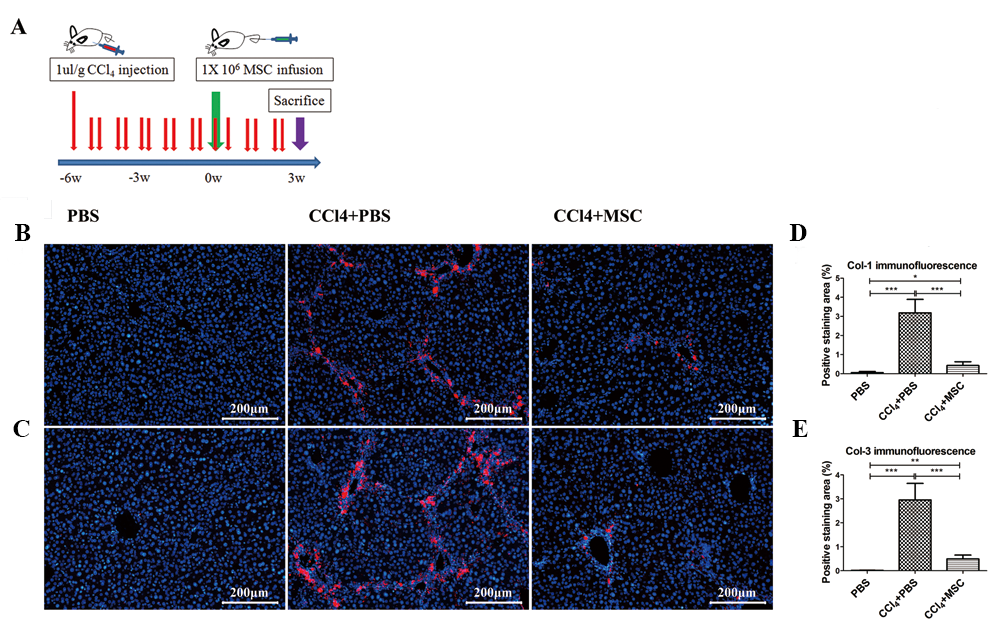


**Supplementary Figure 5.**MSCs therapy down-regulate macrophages and convert CD4+ helper T lymphocytes system of the body into an anti-inflammatory condition in TAA-stimulated spleens. MSCs infusion down-regulates **(A)** Th1, **(B)** Th17 cells and **(E)** macrophages and up-regulates **(D)**Treg cells at 24h or 48h or 72h after MSCs transplants for TAA-stimulated mice. The marked change of **(C)** Th2 was not observed after MSCs transplants.


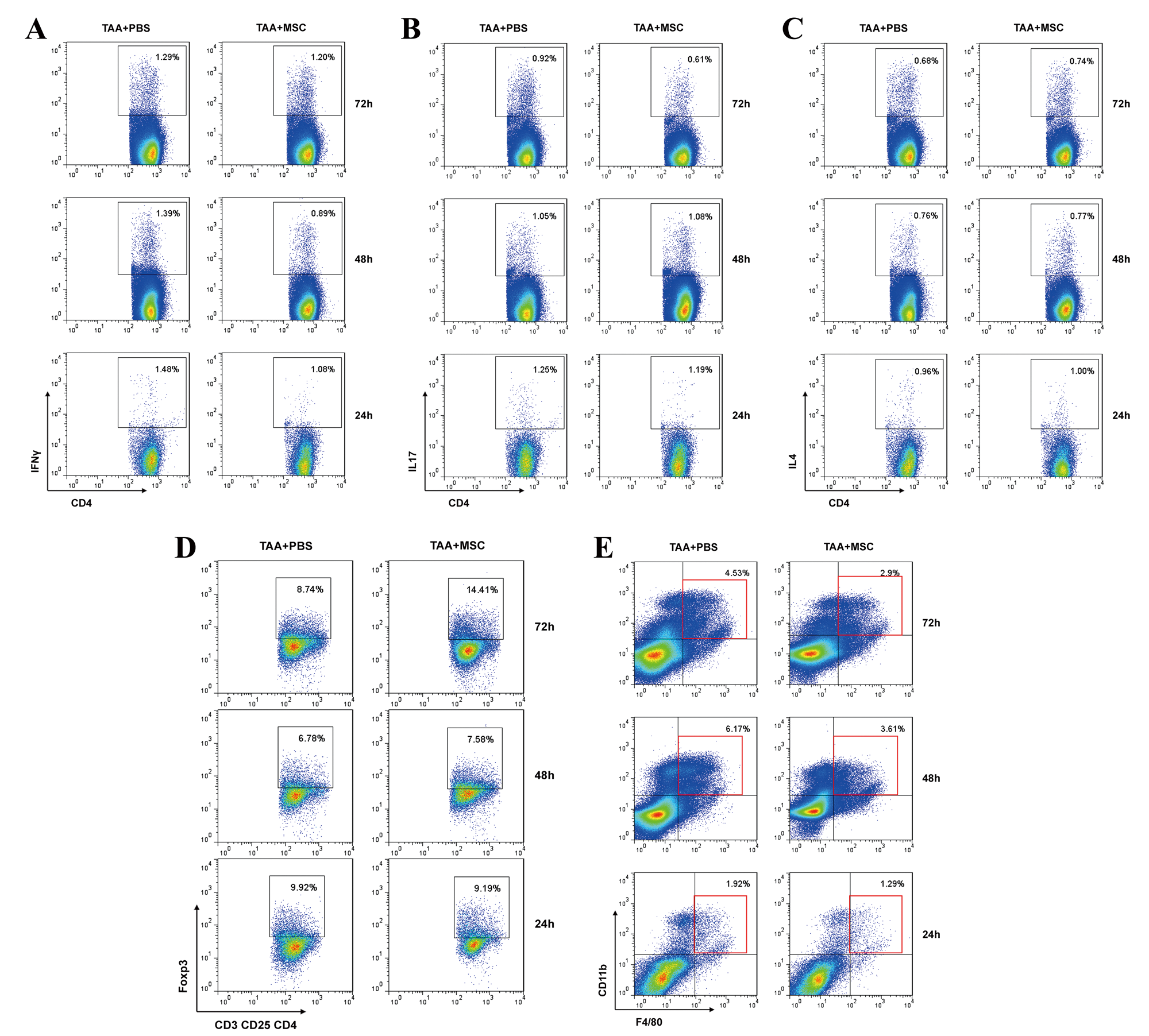


1 Ming, Z., Fan, Y. J., Yang, X. & Lautt, W. W. Contribution of hepatic adenosine A1 receptors to renal dysfunction associated with acute liver injury in rats. *Hepatology***44**, 813-822 (2006).

2 Bernal, W., Auzinger, G., Dhawan, A. & Wendon, J. Acute liver failure. *The Lancet***376**, 190-201 (2010).

3 Parekkadan, B. *et al.* Mesenchymal stem cell-derived molecules reverse fulminant hepatic failure. *PloS one***2**, e941 (2007).

4 Krizhanovsky, V. *et al.* Senescence of activated stellate cells limits liver fibrosis. *Cell***134**, 657-667 (2008).

5 Bataller, R. & Brenner, D. A. Liver fibrosis. *Journal of clinical investigation***115**, 209 (2005).

6 Singer, N. G. & Caplan, A. I. Mesenchymal stem cells: mechanisms of inflammation. *Annual Review of Pathology: Mechanisms of Disease***6**, 457-478 (2011).

7 Dayan, V. *et al.* Mesenchymal stromal cells mediate a switch to alternatively activated monocytes/macrophages after acute myocardial infarction. *Basic research in cardiology***106**, 1299-1310 (2011).
